# Dissertation on the Morbid Sympathies between the Mouth and Other Parts of the Body

**Published:** 1843-09

**Authors:** Thomas E. Bond

**Affiliations:** Professor of Pathology and Therapeutics in the Baltimore College of Dental Surgery.


### ARTICLE II.

*Dissertation on the Morbid Sympathies between the Mouth and other parts of the body.* Read before the American Society of Dental Surgeons, at its fourth Annual Meeting; Baltimore July 19th, 1843. By Thomas E. Bond, Jr. M. D. Professor of Pathology and Therapeutics in the Baltimore College of Dental Surgery.

The body of every animal is wisely contrived and perfectly fitted for the purposes it is intended to subserve. Every part, however minute, is necessary to the complete performance of the work of the whole machine, and a beautiful unity of purpose and dependence of parts is observable throughout the entire organization. So remarkable is this unity and dependence, that a naturalist, by examining a fragment of any one of the bones of an animal, may at once determine the character of the individual it represents. Because, having ascertained the size, figure, &c. of any one bone, he may reason with infallible certainty, that every other part of the body to which it belonged, was formed in perfect proportion to this part, and with strict reference to the purposes for which this particular portion was designed. Should a naturalist ascertain that a single bone presented to him, was constructed for purposes of prey, he would immediately infer that a beast so provided must have had strong muscles and bones of the neck, and jaws to enable it to hold and tear its prey, hind legs of such formation as to enable it to spring upon it, claws to seize and hold it, and a digestive apparatus suited to the reception and assimilation of food thus procured. In short a body is a unit, and though for the sake of description we speak of its several parts, it is a great whole, fed by one aliment, nourished by one blood, vitalized by one nervous system, directed to one purpose and obeying one general law of sustentation, decay and death. Those who are familiar with the writings of Cuvier, will not need to be reminded of his beautiful reasoning upon this subject. Now as one part of the body is so identified with the others, it is necessary that a certain organic understanding (so to speak) shall exist between the several parts, in order to enable them to act in concert, in carrying on the business of life. For instance, in the human body, the *eyes* must act together; the *muscles* of the *trunk* must aid those of the *limbs,* and many other agreements of motion must subsist between the distant parts of the frame. *Then* again, the body has not only to perform certain primary works, by which it may be continued in being, &c. but being liable to injury, and being constantly assailed by enemies from without and within, it must have certain signals of suffering, and arrangements of action, by which any part more particularly attacked may receive succor from the rest. *Again,* being connected together by extension of tissues, or by the intervention of their lamina of other structures, by blood vessels and by nerves, morbid conditions of one part are necessarily propagated to others—all this concert of parts whether physiological or pathological is called sympathy—and constitutes one of the most interesting and beautiful peculiarities of organized structures. In many instances the dependence of one part upon another is so direct, and the mode of communication so evident, that there is no difficulty in detecting the process of sympathetic action or suffering that may be observed between them. In other cases this concert of parts depends upon undiscovered links of union, and is known to exist only upon the evidence of common observation.—Besides this sympathy of parts, there is a general interest of the whole organism in the welfare of all its parts, and severe or long continued suffering in any one however relatively unimportant will commonly create a general disturbance of health, and may involve the whole frame in serious and even fatal disorder.

Although in many cases no change in the structure of morbidly sympathizing parts might be discovered upon autopsic observation, yet there is good reason to believe that sympathy of this kind is in fact a transfer or propagation of actual molecular change—and every physician knows that a disorder, primarily of little importance may prove fatal by involving vital organs in a sympathy of disease.

It cannot, therefore, be predicated of any organ that its suffering is necessarily unimportant to the health of the whole system; since *experience* shows that the danger of almost all disorders, depends very much upon the sympathies likely to be established in the course of their progress—and the importance of these sympathies is not always regulated by that of the organ originally involved.

In compliance with your request I will proceed to comment, in that brief manner which the nature of the occasion requires, upon the morbid connexions of the dental structures with other parts of the body.

The subject naturally resolves itself into two parts.

1st. It will be necessary to inquire how far and in what way the particular structures in question are influenced by the disorders of other parts. 2d. How far and in what way other parts are influenced by diseases of the dental arch and appendages.

The teeth, are affected by all the disorders that interfere extensively with the complicated process of assimilation—scrofula, scorbutus, and indigestion, from whatever dyspeptic cause, are sure to produce more or less disease in the dental apparatus. The first of these, scrofula, being often inherited, and developing its mischievous nature very early in life, influences the teeth during the important process of formation and by preventing their perfect organization, renders them unable to resist the influences of morbific causes, which, otherwise, might not be sufficient to cause decay. Unhappily this terrible disorder is most commonly inherited and is generally incurable—nevertheless much may be done to prevent the rapidly fatal encroachments of scrofula upon vital organs, by the prompt and persevering use of hygienic means. It is not at all likely, however that any remedies of this kind can improve the feeble organization of the teeth, and therefore the skill of the dentist in these cases must be directed to the removal of agents capable of destroying the vitality of these organs, and to the mechanical repair of damage, already done.

*Scorbutus or Scurvy.*—This extraordinary affection, the pathology of which is not well understood, seems to be connected with, and perhaps to consist in such a vitiated condition of the blood as renders it unable to supply the wants of any part of the body. While every tissue and organ gives signs of suffering, the gums especially, become swollen, livid, soft and spongy. They are liable to bleed at the slightest touch, sometimes become deeply ulcerated and not unfrequently the teeth drop out.

The causes of scurvy are such as seldom operate upon other subjects than seamen or prisoners. For a long time it was the scourge of navies, but now, owing to the provision made against it, it rarely produces much destruction of life. The treatment of this disease can hardly be considered proper to dental surgery.

Dyspepsia or indigestion, expresses only the mal-performance of an act which is the result of the combined effort of various organs. Therefore, as failure of function in any one will interrupt the healthy performance of the great common object, dyspepsia must be a general term comprising several disorders. Being immediately connected with the digesting apparatus, and in fact forming an important part of that great and complicated system by which aliment is received and prepared for assimilation, the dental apparatus can hardly escape injury when the other organs of this system are involved in suffering. Indeed the mucous membrane, which in the stomach and intestine is the seat of digestion, and in the mouth is continually pouring out important fluids from its surface and glands, is so intimately connected with the dental arch as to unite it in close sympathy with the more important organs of alimentation—a healthy state of the fluids of the mouth is necessary for the safety of the teeth, and it is not to be expected that the secretions of the mouth will be healthy, when the functions of the intestinal membrane are disturbed. Every dentist must know that it is a hopeless task to attempt to save the teeth from caries, unless the patient can be saved from dyspepsia.

*Syphilis* also, by vitiating the general glandular and secretory systems, produces such a state of fluids as may rapidly destroy the teeth.

*Rickets* very much delays dentition, and the teeth when protruded are liable to rapid decay.

The enamel of the permanent teeth is often craggy and worm-eaten (in appearance) showing its imperfect formation, though sufficiently hard. The fang during the progress of the disease, has been found somewhat softer than natural.

Febrile diseases, especially the exanthemala, influence the perfection of dentition. Measles very often leaves evidences of its visit upon the enamel of the teeth, in the pitted appearance which they present. As fever is always attended with vitiation of the secretions of the mouth, we may readily perceive how any protracted disease of this kind may injure the teeth.

All serious diseases of the antrum, must involve the dental arch—inflammation may be propagated, nutrition impeded, caries communicated, and the arch actually broken up in the course of those often fatal diseases which have their seat in this important cavity. The surgeon dentist should be well acquainted with the various disorders and morbid growths which may be developed in the antrum. Early detection is often necessary in order to cure, and none is so likely to have the opportunity of discovering the hidden mischief as the dentist. The first symptoms of disorder are often felt in the teeth, and unless the dentist who may be consulted is able to point out the true nature of the evil, delay may be occasioned, and delay may be fatal.

Mercurial salivation (ptyalism) has often caused extensive devastation in the dental arch. Mercury, like every other gift of God, has been shamefully abused, and serious and even fatal injuries have resulted from the reckless administration of this most useful medicine. Unhappily, the occurrence of such calamitous accidents has induced such general and unreasonable prejudice against the use of mercurial medicines, that vastly more injury has been suffered by society by improperly withholding, than by the most reckless abuse of them. The feeling against mercury has been the common hobby horse of charlatans, and of unprincipled physicians, aud it needs no little firmness to enable a practicien to deal honestly with his patients in the use of this drug. From the fact that salivation is injurious to the teeth, dentists have been led to comment severely on the use of calomel, and they have done much to spread abroad terrible notions of the evils inseparable from the employment of this and other mercurial preparations. Some of these gentlemen have seen evidences of mercurial devastation in every form of disorder and variety of decay, presented to their observation, and to such, calomel is the one thing dreadful to all who live to eat, and eat to live.

I am well aware that salivation, especially if profuse, must be destructive to the teeth, and fortunately it is at length understood that ptyalism is not by any means necessary to the attainment of all the benefit of mercury. Salivation is an accident always to be dreaded, and as far as possible avoided, yet even at the risk of it, mercurial remedies are often indispensable; inasmuch as life is more important than teeth.

There is no reason to believe that the use of mercury in injurious to the teeth, when salivation is not induced. Yet very often caries of these organs is most unjustly attributed to it. People are exceedingly apt to confound the post hoc, with the propter hoc, and dentists are as liable as other men to fall into this error. A patient who has escaped a severe attack of fever finds his teeth rapidly decaying. In great alarm he applies to the dentist, the latter examines the mouth, and asks his patient if he has been taking calomel—he replies that he has, and then the man of science, as at least he ought to be, breaks forth into a stereotyped and entertaining diatribe against mercury as the origin of all the evil? And why, might it not be just as properly attributed to the magnesia or tartar emetic the patient had taken?—The fever itself was sufficient cause for all the resulting evil, why then transfer the blame to the remedy by which that fever was subdued, and throw implied and perhaps ruinous censure upon the physician whose judicious use of this very drug has perhaps saved the patient's life.

Until I have other information than I now possess I cannot believe that the proper employment of mercury is injurious, and while I reprobate its abuse, and would consider the physician unpardonable, who would be careless or reckless in the use of a medicine capable of doing so much harm, I cannot but regard that man as the author of greater evil, who, by silly declamation against an important remedy, fetters the physician in his contest with the most formidable disorders.

Having thus rapidly glanced at several forms of disease which more or less affect the teeth, we will now, with equal brevity, enumerate some of the evils which may be inflicted upon other parts of the body by disorders of these organs.

And first we may remark that the teeth like other organs may propagate disease to adjacent tissues, or may, by becoming foreign bodies, produce disorder in neighbouring parts. By a wise and beautiful provision of Providence, parts which cease to be useful are thrown off from the body by the action of adjacent parts. The dead portion becomes by the mere fact of its loss of vitality, an irritant to the very surfaces with which when in its normal condition it was placed in comfortable contact. It is evident that this irritation will be prolonged in proportion to the tardiness with which the foreign body is removed, and as the teeth require great length of time for their removal, the irritation produced by these when in condition to irritate, must be long continued.

Disease may be propagated too from the inflamed or ulcerated fangs to the lining membrane of the antrum, and thus the teeth may become the first cause of a series of morbid events, ending perhaps in fatal consequences. In the same way disease may be communicated from one tooth to another.

The irritation produced by the teeth may produce local or general disorder.

The *local* consequences may be either immediate, such as we have just described, or they may be produced at points more or less distant.

Among the first the most common is inflammation and abscess in the gums, and cellular tissue between the gum and cheek, next to these perhaps are some morbid condition often undiscovered, of the lining membrane of the antrum. Among the second class of local consequences we notice neuralgic affections of various parts of the face.

It is very important that painful affections of the nerves of the face should be traced to their true origin. Most cases of neuralgia that occur in these parts are not at all dependent upon dental irritation, but upon constitutional causes, which require general remedies. They are, for the most part, either intermitting neuralgias, originating from marsh poison, or symptomatic nervous affections, dependent upon general atonic conditions.

It is highly important that the dentist be able to discriminate these disorders well; so that he may act with judgment, either in removing teeth or saving them, as they may or may not be the cause of evil. Dyspeptic conditions may also arise from disordered teeth, as dental disorder may arise from dyspepsia. Sympathies cannot be one sided, and as the teeth suffer from a disordered stomach, so the stomach will suffer from disordered teeth.

When extensively diseased the teeth and gums pour out putrid matters which poison the saliva and unfit it for its purpose in digestion. Moreover the teeth are not able to masticate the food and it is sent into the stomach unprepared for being acted on by the solvent fluids. Besides, nothing is more sure to disorder the stomach than long continued pain, and when the teeth are a mass of disease, the patient is rarely exempt from suffering.

I have known dyspepsia and general ill health, amounting to entire disqualification for the business of life, to depend upon carious teeth, and to be removed by treatment addressed to these organs.

The process of digestion requires a concentration of nervous energy, and cannot be properly performed if pain calls off the nervous attention to another part. Therefore, for this reason as well as for others, pain is, of itself, a serious evil to health, and wherever located, if long continued it will destroy it.

If the teeth were otherwise unimportant, as organs capable of suffering much and long continued pain, they would be well worthy of the special attention of skilful surgeons.

But the teeth may originate general disorder. This may be effected as the consequence of the local disturbances, above alluded to, becoming of sufficient importance to involve the entire economy. Symptomatic fever may be set up as the result of local imflammation, or general functional debility may evince the imperfection of digestion. The nervous centres are also, under certain circumstances, so fearfully impressed by dental irritation as to convulse the remotest fibres of the frame, and even to overwhelm the powers of life.

It is very remarkable that these extensive and often fatal consequences do not result from diseased but from healthy states of the dental system. The regular growth of the teeth is frequently attended with most disastrous consequences.

The well known evils of first dentition, seem to depend upon the simple fact, that, this process causes prolonged suffering. The pain is the prime cause of all the mischief, though certainly the condition of the nervous and vascular systems which accompany the evolution of these teeth, forms a very necessary part of the array of circumstances which unite to produce such formidable results. The very first symptom of severe dentition is found in the vitiated condition of the alvine discharges, resulting from irritation of the intestinal canal—extended perhaps to all the secreting organs connected with it. This fact points out the sympathy which exists between the mucous membrane of the mouth and its prolongation into the intestines. Very often, however, the brain and spinal marrow become most seriously interested; fearful convulsions attest the universal sympathy of the frame, and not unfrequently death results from the apparently trifling irritation produced by the pressure of a single tooth upon its gums.

And now, gentlemen, the simple inference which I would draw from the facts I have so rapidly stated is, that as the teeth are important parts of the body; and as the body is a unit—knit together by the closest ties, pervaded by one system of blood-vessels and nerves, and directed in the performance of its functions by an essential law of sympathy; as diseases of other organs affect the teeth, and conversely, the diseases of the teeth affect other organs—*therefore,* the treatment of the teeth is properly a branch of medicine, and none can be supposed to be qualified for it who are not acquainted at least with the elementary principles of medical science.

